# A web-based psychological support program for caregivers of children with rare chronic diseases: a randomized controlled trial

**DOI:** 10.1186/s13023-024-03029-9

**Published:** 2024-01-27

**Authors:** Dunja Tutus, Mandy Niemitz, Paul L. Plener, Jörg M. Fegert, Christine Lehmann, Christa Weiss, Christine Knaevelsrud, Lisa Biehl, Miriam Rassenhofer

**Affiliations:** 1 Department of Child and Adolescent Psychiatry/Psychotherapy, Ulm University, Steinhoevelstr, 5, 89075 Ulm, Germany; 2 Department of Child and Adolescent Psychiatry, Medical University of Vienna, Vienna, Austria; 3 Department of Paediatric Respiratory Medicine, Immunology and Critical Care Medicine, Charité University Medicine Berlin, Berlin, Germany; 4 Department of Education and Psychology, Free University of Berlin, Berlin, Germany; 5 German Alliance of Chronic Rare Diseases, Berlin, Germany

**Keywords:** Internet-based cognitive behavioural therapy (iCBT), Parental psychological stress, Randomized controlled trial (RCT), Rare chronic diseases in childhood and adolescence, Web-based psychological support programme for caregivers of children with rare chronic diseases (WEP-CARE)

## Abstract

**Background:**

Approximately 50% of rare diseases have symptom onset during childhood. A high level of nursing care and an often uncertain prognosis put caregivers of the affected children at high risk for psychological distress. At the same time, their caregivers have limited access to appropriate psychological care. The aim of this study was to evaluate a web-based psychological support program for caregivers of children with chronic rare diseases (WEP-CARE).

**Methods:**

German-speaking parents (recruited between May 2016 and March 2018) caring for children aged 0–25 years with a rare disease showing clinically relevant anxiety symptoms, were assigned to either the WEP-CARE (*n* = 38) or treatment as usual (*n* = 36) condition within a randomized controlled trial. The primary outcome measure was parental anxiety, assessed with the Generalized Anxiety Disorder Questionnaire (GAD-7). Secondary outcomes were fear of disease progression, depression, coping, quality of life and user satisfaction. The group differences were tested through repeated-measures analyses of variance. The WEP-CARE group was additionally followed up three months after the treatment.

**Results:**

A significant time-group interaction was found for anxiety (*F* (1,35) = 6.13, *p* = .016), fear of disease progression (*F* (1,331) = 18.23, *p* < .001), depression (*F* (1,74) = 10.79, *p* = .002) and coping (*F* (1,233) = 7.02, *p* = .010), suggesting superiority of the WEP-CARE group. Sustainability of the treatment gains regarding anxiety, fear of disease progression and coping was confirmed at the 6-month follow-up assessment (*p* < .01). A significant interaction effect could not be found for quality of life (*F*(1,2) = 0.016; *p* = .899). Both participating parents and therapists were satisfied with WEP-CARE.

**Conclusions:**

Our results underline the efficacy and feasibility of WEP-CARE for parents of children with various rare diseases.

## Background

A rare disease (RD) is a health condition defined by a prevalence threshold, which ranges from 5 to 76 cases in 100,000 people according to different definitions [1]. For example, in the European Union (EU), a disease is classified as RD if the frequency threshold is not more than 50 in 100,000 people [1, 2]. To date, between 5000 and 8000 distinct RDs have been documented [3], whereas new RDs are reported regularly in the medical literature [1]. Therefore, the number of patients affected by RDs is large, 300 million people worldwide (approximately 30 million people in the EU, 25 million in the United States), or approximately one person out of 15, which makes RDs a global health issue [3, 4]. Approximately one out of two RDs have onset during childhood [5]. RDs represent a very heterogeneous group, comprising many different disease-specific entities, with mostly complex, severe, degenerative and systemic, chronic debilitating, or life-threatening disease patterns, frequently associated with disability and reduced life expectancy [2, 5]. RDs usually show a progressive course and are rarely curable, since approximately 80% of them are at least partially genetically determined [5]. Health care, as well as carrying out scientific studies, is usually difficult due to the small number of patients with a certain RD and limited number of experts scattered nationwide or across countries [5]. Some RDs are extremely rare, meaning that basic knowledge, including etiology, pathophysiology, natural course of the disease and epidemiological data, is limited or missing [3]; hence, specialized facilities, reliable diagnostic procedures and standardized treatment protocols are often not available [5]. On the one hand, some RDs are associated with unspecific symptoms that can be different for each individual patient; on the other hand, many symptoms overlap with more common disorders, thus making diagnosis problematic [5]. Due to their rarity, most physicians will never hear of most RDs, and even fewer have a chance to diagnose an affected patient [3]. Many patients have to obtain information about disease-specific treatments and specialized medical centres on their own, which is frequently associated with additional financial burden [5, 6]. As a rule, making a reliable clinical diagnosis is delayed and takes on average approximately seven years [5]. During this odyssey, the patients have to undergo numerous medical examinations, clinic appointments and inpatient stays, which lead to an inefficient use of resources [5]. A severe loss of life expectancy and quality of life (QoL) are common consequences of late or incorrect diagnosis [5].

Confrontation with the diagnosis, frequently with uncertain prognosis of the disease, or long-lasting complaints without a clear diagnosis, are associated with high emotional stress for parents and worries about the child´s well-being and future prospects [6, 7]. Following the diagnosis, many parents experience a phase of relief, guilt, uncertainty, and disbelieve, culminating in emotional shock, often manifested in the form of an acute stress reaction [8, 9]. Depending on the diagnosis, the parents may experience fear of losing the child [7]. Since a great number of RDs occur from birth and are often severe, many of them require a very high level of care, including managing multicomponent, sometimes invasive, frequently complex, and time-consuming treatment regimens [6, 9, 10]. Moreover, many families have to deal with uncertainty about the individual clinical course of the disease, a lack of appropriate therapy, frequent hospitalizations, school or/and work absenteeism, restrictions of activities (due to physical impairment or the medical regimen), residual symptoms, persisting functional restrictions, and late sequelae for patient psychosocial development, which are associated with substantial burdens [6, 9]. Parents must balance the treatment plan, professional obligations, personal needs and needs of other family members [6, 10]. In some cases, the caregivers can no longer work, which represents an additional financial burden [5]. This means that many patients with chronic RDs and their caregivers face a variety of problem areas, and those affected often have a strong need for extensive psychosocial care [5, 6]. Children suffering from RDs and their parents report not only increased psychological strains (characterized by tension, restlessness, confusion, disbelief, uncertainty, insecurity, helplessness, excessive demands, fears, worries, concerns and the feeling of loneliness) but also loss of confidence in the health care system and the medical profession [3, 5, 6, 11, 12]. Some parents of children with long-term and genetically determined conditions experience chronic sorrow and feelings of self-blame due to an inability to accept the child´s disease, continually searching for reasons for their child’s condition [8, 9, 12]. There is consistent evidence that affected patients and their parents show an increased risk of mental health problems such as anxiety and depression [13, 14]. Fear of disease progression is frequently described in patients with chronic somatic conditions or their family members [15]. Disease-specific factors, such as disease severity, child’s physical functioning and pain, hospitalization, and infection, are associated with lower parental QoL [16]. Parental mental health problems are associated with problems in daily functioning, management of the disease and therapy adherence, which may have a negative effect on the course of the disease [9, 10, 14]. Quittner et al. [13] found that parental depression or anxiety double the risk that the affected child would report elevated psychological distress. Parental disease-related fears and inadequate coping mechanisms may contribute to problems in communication, conflicts with an adolescent child and his or her desire for autonomy and the child´s feeling of hopelessness, which in turn can negatively affect the course of the disease [9, 11]. Effective disease management requires a multidisciplinary approach, including appropriate medical and pharmacotherapeutic treatment, therapeutic patient education, and adequate psychosocial support to help patients and their families cope with an RD as well as possible [5, 17]. Improving the infrastructure for medical and psychosocial care for RDs could diminish the burden of disease for many patients and their families [3]. Psychosocial support is even more crucial in the case of RDs without available evidence-based treatment options [3].

However, many parents in need do not seek conventional psychosocial care because this would require additional time and emotional resources [6, 12, 18]. Although children’s hospitals in Germany offer psychosocial care in the form of consultation, evidence-based and comprehensive treatments for affected parents are barely available [18]. Long waiting times for standard psychotherapeutic care in Germany are another obstacle for affected parents to obtain the required treatment [19]. Besier and Goldbeck [20] found that only 13.6% of parents with clinically elevated anxiety and/or depression caring for a child with cystic fibrosis (CF) received psychopharmacological and/or psychotherapeutic interventions, whereas 30.9% were in contact with the psychosocial service of their child’s CF unit in Germany. In summary, barriers to caregivers’ psychosocial treatment comprise long distances to children’s hospitals, restricted time capacities to care for themselves, or less awareness of one’s own well-being compared to the well-being of the child [21]. However, early and timely intervention can limit psychological distress and have an encouraging influence on the wellbeing of caregivers [8]. A growing body of research suggests that web-based interventions have the potential to overcome the abovementioned barriers, increase the coverage of usual care services, and reach people who cannot be reached otherwise, thereby delivering effective psychological treatment [22, 23]. Internet-based cognitive behavioural therapy (iCBT) seems to be as effective as conventional cognitive behavioural therapy for several psychiatric disorders [23]. The large effect sizes and the limited therapist time required suggest that iCBT is highly cost effective for anxiety disorders and depression [22, 23]. However, there is a lack of iCBT interventions designed to address parental psychological problems precipitated by children´s chronic somatic conditions, or such interventions, if available, need further evaluation [19]. A web-based psychological support program for caregivers of children with chronic RDs (WEP-CARE) [21], designed as guided iCBT writing therapy, was found to be feasible and promising regarding its efficacy in improving mental health and QoL in parents caring for a child with CF.

The first aim of this study was to further evaluate WEP-CARE within a sample of parents caring for children suffering from a wide range of different chronic RDs. Since the efficacy of WEP-CARE was investigated so far only within a pilot study, the second aim of this study was to compare WEP-CARE with Treatment-As-Usual (TAU) within a randomized controlled trial (RCT). Therefore, we hypothesized that WEP-CARE is superior to TAU regarding the reduction in parental generalized anxiety symptoms, fear of disease progression, depression and improvement of parental coping and QoL. We expected to find superiority of WEP-CARE at posttreatment, or to be precise at the 3-month follow-up (3MFU), and sustainability of all treatment gains three months after the end of the program at the 6-month follow-up (6MFU). Finally, the third aim was to explore parents´ and therapists´ satisfaction with the program.

## Methods

### Design

The RCT participants were recruited between May 2016 and March 2018 in cooperation with the German Alliance of Chronic Rare Diseases (ACHSE) [24]. Referrals by clinicians treating children with RDs (e.g., in CF outpatient clinics and centres for RDs) throughout Germany and self-referrals of the study participants were encouraged. Additionally, directly approaching support groups, parents’ initiative and forums on the internet (e.g., Facebook) by the project team appeared to be a successful strategy for reaching potentially eligible families. The caregivers interested in study participation had to register at the website Ulm Online Clinic (UOC; https://ulmer-onlineklinik.de/) in the first step [25]. After confirming their registration, the caregivers were invited to fill out questionnaires online. Those who met all inclusion criteria were invited to participate in the study. Caregivers eligible for study participation were randomly assigned to either the WEP-CARE or TAU condition.

### Participants

The study sample consisted of *N* = 74 German-speaking parents caring for a child with RD or suspected RD. Further eligibility criteria were as follows: child´s age 0–25 years; parental clinically elevated anxiety symptoms, defined by a raw symptom score ≥ 7 in the Generalized Anxiety Disorder-7 (GAD-7) [26, 27] questionnaire; internet access during study participation; and sufficient knowledge of the German language. Exclusion criteria: acute suicidality and psychotic symptoms were examined as part of the online screening using the Patient Health Questionnaire (PHQ-9) [28, 29] and the General Perceptions List [30] and via phone interviews with licensed psychotherapists, if required.

The most frequently reported children´s RDs in our sample were: CF (*n* = 10; 13.5%), suspected VACTERL association (*n* = 5; 6.8%), Prader-Willi syndrome (*n* = 4; 5.4%), neurofibromatosis, hypopituitarism and spinal muscular atrophy (each *n* = 3; 4.1%). Further children´s RDs in our study sample were: syringomyelia, tuberous sclerosis, Ehlers–Danlos syndromes, Pompe disease, macrocephaly-capillary malformation, Crouzon syndrome, ectodermal dysplasia, MECP2 duplication syndrome, metachromatic leukodystrophy, osteogenesis imperfecta, Fabry disease, GLUT1 deficiency, adenylosuccinate lyase deficiency, Goldenhar syndrome, Williams–Beuren syndrome, osteopetrosis, opsoclonus myoclonus syndrome, Smith–Magenis syndrome, Becker muscular dystrophy, distal 18q-, Pitt–Hopkins syndrome, connective tissue disease, hypoplastic left heart syndrome, protein losing enteropathy, juvenile idiopathic arthritis, tetralogy of Fallot, aqueductal stenosis, Rett syndrome, Sotos syndrome, diaphragmatic hernia and osteopathia striata with cranial sclerosis. *N* = 7 participating parents reported more than one RD.

**Fig. 1:**
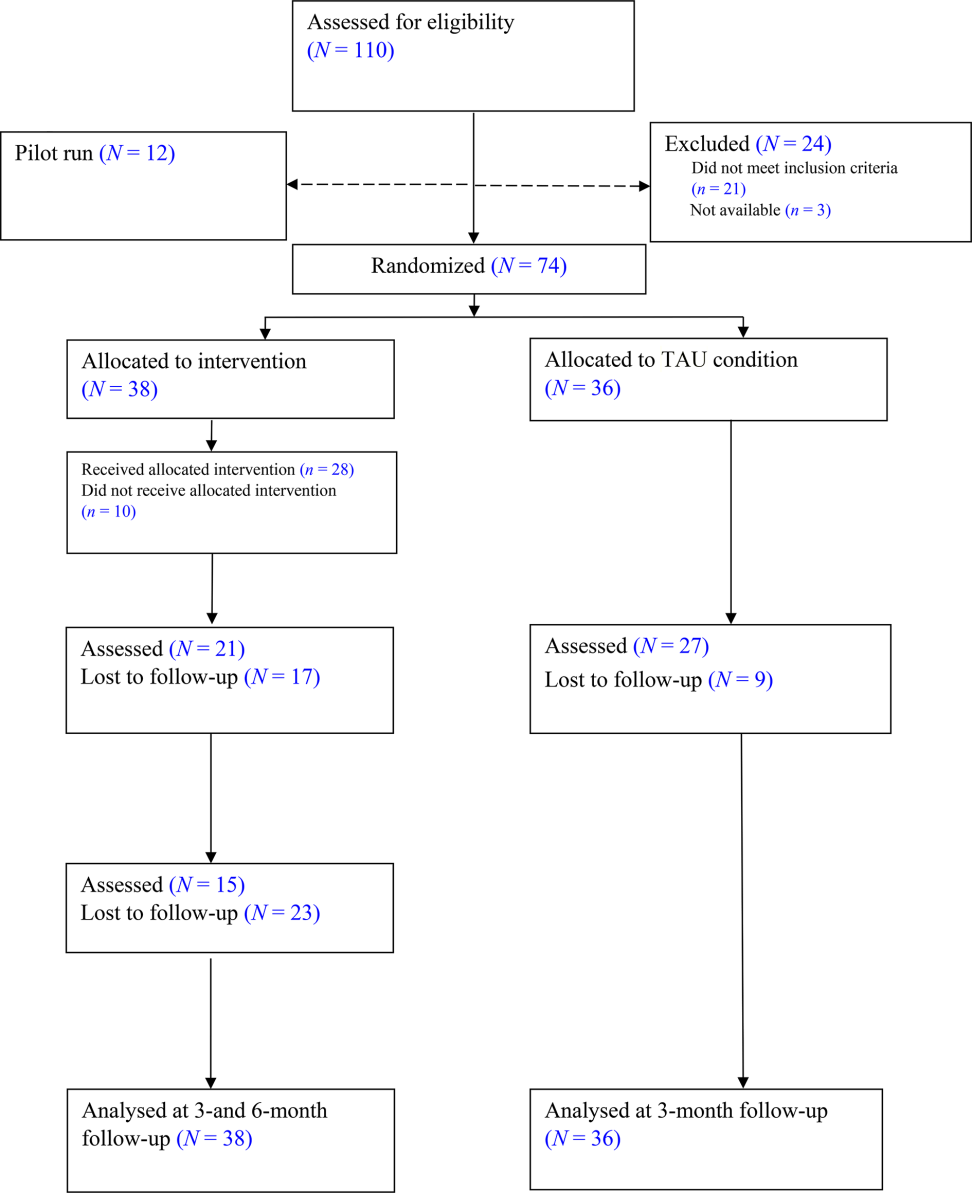
CONSORT flowchart of the study

Overall, *n* = 8 (10.8%; four in each condition *p* = .936) of the participating parents reported undergoing another form of psychosocial support/psychotherapy, and *n* = 7 (9.5%; TAU = 5; WEP-CARE = 2; *p* = .503) were on psychotropic medication (antidepressant and/or anxiolytics) at the time of the baseline assessment. Only one participant (WEP-CARE condition) reported a change in the type of medication *p* = .484), and two participants (TAU condition) reported a change in the in dose of the medication *p* = .523) within a timeframe of four weeks before the study admission. Participating parents were randomly assigned to either the WEP-CARE (*n* = 38) or TAU (*n* = 36) condition within the RCT (see Fig. 1). The sociodemographic characteristics of the parents and their children are displayed in Table 1.

**Table 1:** Sample description of participating parents and their children in the study at baseline

	Total *N* = 74 (100.0%)	WEP-CARE *n* = 38 (51.4%)	TAU *n* = 36 (48.6%)	Statistics
**Parents**				
Gender: female *n (%)*	66 (89.2)	36 (94.7)	30 (83.3)	*χ²*(1) = 2.49 *p* = .114
Age in years *M (SD)*, min - max.	40.18 (7.65) 26–57	39.89 (7.38) 26–56	40.47 (8.02) 26–57	*t*(72) = − 0.32 *p* = .748
Completed educational/vocational training, or university education *n (%)*	73 (98.6)	37 (97.4)	36 (100.0)	*χ*²(1) = 0.96 *p* = .327
Current employment *n (%)*				*χ*²(3) = 7.43 *p* = .059
Non-working	19 (25.7)	12 (31.6)	7 (19.4)	
Full-time employed	13 (17.6)	3 (7.9)	10 (27.8)	
Part-time employed	31 (41.9)	19 (50.0)	12 (33.3)	
Occupational retraining or marginally employed	11 (14.9)	4 (10.5)	7 (19.4)	
Living together with a partner: *n (%)*	60 (81.1)	32 (84.2)	28 (77.8)	*χ²*(1) = 0.50 *p* = .480
GAD-7 *M (SD)*, min - max.	11.78 (4.19) 7–21	11.53 (3.90) 7–21	12.06 (4.52) 7–21	*t*(72) = − 0.54 *p* = .591
FoP-Q-SF *M (SD)*, min - max.	40.04 (7.37) 25–56	39.61 (7.34) 25–56	40.50 (7.49) 27–56	*t*(72) = − 0.52 *p* = .605
PHQ-9 *M (SD)*, min - max.	10.78 (4.60) 2–22	10.74 (4.89) 2–22	10.82 (4.35) 3–20	*t*(72) = − 0.08 *p* = .937
*ULQIE *M (SD)*, min - max.	47.78 (13.55) 11–82	48.43 (14.29) 11–82	47.09 (12.89) 17–73	*t*(70) = − 0.42 *p* = .676
CHIP-D *M (SD)*, min - max.	23.83 (10.60) 1–45	23.52 (10.41) 1–45	24.16 (10.95) 3–44	*t*(72) = − 0.26 *p* = .794
**Children and adolescents**				
Gender: female *n (%)*	30 (40.5)	16 (42.1)	14 (38.9)	*χ²*(1) = 0.08 *p* = .778
Age in years *M (SD)*, min - max.	6.94 (6.00) 0–24	6.68 (6.42) 0–24	7.21 (5.61) 0–17	*t*(72) = − 0.37 *p* = .710
Rare diseases *n (%)*				*χ²*(3) = 3.99 *p* = .263
Metabolic diseases	20 (27.0)	8 (21.1)	12 (33.3)	
Congenital malformations	16 (21.6)	11 (28.9)	5 (13.9)	
Not specified chromosomal abnormalities	15 (20.3)	6 (15.8)	9 (25.0)	
Other rare diseases	23 (31.1)	13 (34.2)	10 (27.8)	

*Note*: WEP-CARE = a web-based psychological support program for caregivers of children with chronic rare diseases; TAU = Treatment-As-Usual; GAD-7 = Generalized Anxiety Disorder; FoP-Q-SF = the Short Form of the Fear of Progression Questionnaire; PHQ-9 = the Patient Health Questionnaire; *ULQIE = the Ulm Quality of Life Inventory for Parents, raw scores were transformed linearly into a scale ranging from 0 to 100; CHIP-D = the Coping Health Inventory for Parents

Information on the child´s rare disease (RD) was collected via open question and classified based on parental answers. If more than one RD pro child was reported, only the first one is represented in the table. The classification schema was made ad hoc and serves solely for the data description

### Randomization

A biostatistician at the Institute of Epidemiology and Medical Biometry of Ulm University performed the randomization. The ROM software program was used to generate the random allocation sequence. The group allocation of the participating parents took place centrally by the consulting biostatistician, independently of the research group responsible for the study.

### Intervention: WEP-CARE

WEP-CARE is a brief manualized cognitive behavioural intervention, delivered in the form of an internet-based writing therapy, developed to support parents caring for chronically ill children and adolescents while addressing their psychosocial disease management [10]. It aims at reducing parents’ psychological complaints, such as anxiety, fear of disease progression, depression, and/or stress symptoms, and enhancing QoL, mental well-being, and their ability to cope with disease-related stressors [10, 18, 21]. Thus, indirect positive effects on the child with an RD and other family members are expected [18]. WEP-CARE comprises 12 writing assignments (i.e., treatment sessions) over a period of 12–14 weeks. One writing assignment per week should be completed within a timely standard of dealing with the topic for approximately 45 min. Caregivers can choose their own schedule to work on writing tasks since communication with a therapist is asynchronous. Participants provided their written responses to the standardized writing assignments and received individualized feedback with further instructions to each delivery by their therapist within two weekdays. This feedback is supervised to guarantee adherence to the intervention manual [10, 18]. The therapists and supervisors are also available in the meantime in case of crises/questions [10]. WEP-CARE consists of five main topics:

1. Preparation (sessions 1–2): introduction, reflection of current appraisal of the child’s disease and identification of desired changes;
2. Coping with anxiety and fear of disease progression (sessions 3–6): addresses anxiety-provoking situations and associated thoughts and feelings, aiming at restructuring the fear-provoking thoughts and developing a new perspective of the situation while thinking about it in a more functional, adaptive and accurate way;
3. Problem-solving module (sessions 7–10): identification of a real-life problem, development of problem-solving approaches, implementation in a daily routine, identification of barriers and development of strategies to address these barriers in the future, if required;
4. Self-care (session 11): identification of own needs and planning enjoyable activities;
5. The last session: summary and integration, serves reflection and integration of the therapy contents and relapse prevention [10, 21].

WEP-CARE uses established cognitive and behavioural techniques, such as exposition, cognitive restructuring, problem-solving training, writing diaries, and resource activation [10]. To date, WEP-CARE has been successfully evaluated in a pilot study within a sample of parents caring for children affected by CF [21] and implemented in a generic adaptation for parents of chronically ill children [10, 18].

Overall, 12 study therapists, aged 39.67 (min – max. 31–51; *SD* = 6.18) years, delivered WEP-CARE during this RCT. All therapists received a two-day personal training course, provided by the developers of the intervention and licensed psychotherapists, with extensive experience in the field of RDs and online psychotherapy. To complete the training, all therapists had to complete three cases under total (100%) and at least two cases under partial (25%) supervision, delivered via UOC in the written form. All WEP-CARE intervention cases during the RCT were supervised at least partially. Additionally, group supervision was delivered via biweekly conference calls. The study therapists treated each 6.08 (min – max. 2–14; *SD* = 4.36) parents on average. The two supervisors were also developers and experienced WEP-CARE therapists, psychologists and licensed psychotherapists with years of practical experience with people affected by RDs. The average number of WEP-CARE sessions for the *n* = 28 participants who started with WEP-CARE (see Fig. 1) was 8.89 (*SD* = 3.86). WEP-CARE completer (*n* = 22), participants who completed at least six sessions, including Coping with anxiety and fear of disease progression module, received 10.59 (min – max. 6–12; *SD* = 2.13) sessions with an average duration of the treatment 148.27 (min – max. 77–418; *SD* = 78.31) days. Among the diverse tested sociodemographic variables, only “living together with a partner” was positively associated with treatment completion in our study sample (*χ*²(1) = 4.97; *p* = .026).

### Control group

TAU condition represented usual care, as typically provided to caregivers of children suffering from RDs in the German healthcare system. The purpose of a 3-month TAU condition was to control for spontaneous changes in the study outcomes and to investigate whether WEP-CARE participants benefit from the intervention more than from usual care since receiving other psychosocial interventions during study participation was acceptable for all participating parents. Utilization of other psychosocial interventions was documented and controlled. All study participants randomized to the TAU condition were informed that they would be offered WEP-CARE after the waiting period, if indicated and desired. Hence, participants in the TAU condition finished their study participation at 3MFU and were not assessed at 6MFU, meaning that the primary outcome assessment for both conditions occurred at 3MFU.

### Outcomes and instruments

The primary outcome measure was parental self-reported anxiety symptom level, assessed with the GAD-7. This questionnaire served for screening eligibility for study participation as part of the baseline assessment. The GAD-7 was used for symptom monitoring and was completed between every WEP-CARE session in the treatment condition, hence 12 times, in addition to the baseline, posttreatment and 6MFU assessments. In the TAU condition, GAD-7 was completed at the baseline, six times serving as symptom monitoring, during the 3-month waiting time, and after the end of the waiting time, at the 3MFU assessment. The GAD-7 comprises seven items reflecting the most prominent features of the DSM-IV diagnostic criteria for generalized anxiety disorder [27]. Hence, it is a valid and reliable (Cronbach’s *α* = 0.84 in our study sample at baseline) screening instrument developed to identify probable cases of generalized anxiety disorder and to assess symptom severity [27]. On the GAD-7, subjects are asked how often, during the last two weeks, they have been affected by each of the seven core symptoms, with response options ranging from 0 to 3, from “not at all” to “nearly every day”. The GAD-7 scores range from 0 to 21, and scores of five, 10, and 15 represent mild, moderate, and severe anxiety symptoms, respectively [27]. The German version of the GAD-7, used in this RCT, was compiled with seven steps of translation and blind back-translation conducted by four independent translators, directed and supervised by one of the developers of the original English version [26].

The following secondary outcomes: fear of disease progression, depression, QoL and coping were also assessed via widely used self-report questionnaires at baseline, 3MFU and 6MFU (in participants in the WEP-CARE group). User satisfaction was assessed with questionnaires developed for the purpose of this study. Participating parents completed the satisfaction questionnaires as part of the 3MFU assessment, whereas a therapist questionnaire was completed after the end of every WEP-CARE intervention. The Fear of Progression Questionnaire short form (FoP-Q-SF) [31] is a reliable (Cronbach’s *α* = 0.79 in this sample at baseline) and valid instrument, adapted for parents of children with RDs [15]. The short form comprises twelve items, answered on a five-point rating scale 1–5 and summed to form a total score between 12 and 60. The cut-off value is 34 for the short form [32]. Depression was assessed by the major depressive disorder module of the German version of the Patient Health Questionnaire (PHQ-9) [29]. The brief depression severity measure comprises nine items, which score each of the operationalization of the nine DSM-IV criteria as “0” (not at all) to “3” (nearly every day). PHQ-9 scores of five, 10, 15, and 20 represent mild, moderate, moderately severe, and severe depression, respectively [28]. The PHQ-9 is a reliable (Cronbach’s *α* = 0.80 in our baseline study sample) and valid measure of depression severity [28]. If the last question: “Thoughts that you would be better off dead, or of hurting yourself in some way?” [28] was answered with any response option with the exception of “not at all” at baseline, a licensed psychotherapist from the study site conducted a phone interview with a potential participant to explore if the person was at risk of committing suicide. None of the interviews yielded suicide risk, so all interviewed participants could be included in the study if the other inclusion criteria were met. QoL was measured by the Ulm Quality of Life Inventory for Parents of Chronically Ill Children (ULQIE) [7], a 29-item questionnaire specifically developed for parents of children with chronic conditions. Parents indicate their well-being and functioning for each item on a five-point rating scale 0–4 with regard to the past seven days. This instrument covers the domains physical/daily functioning, satisfaction with family, emotional stability, self-development, well-being, and a total QoL score. Psychometric properties are good [7]. Cronbach’s alpha at the baseline assessment was acceptable (0.61 for the total score) in the current study. All raw scores were linearly transformed to 0–100 scales. Higher scores indicate higher QoL. Coping was assessed using the scale Maintaining Social Support, Self-Esteem, and Psychological Stability (18 items) of the German version of the Coping Health Inventory for Parents (CHIP-D) [33]. The items referred to coping strategies related to their child’s RD. The parents were instructed to indicate the subjective effectiveness for each item – the specific strategy in managing the child´s disease (with response options 0–3). Psychometric properties have been shown to be good. Cronbach’s alpha in the current RCT study was good, 0.84, at baseline.

### Sample size/power calculation

The sample size was calculated using the “G-Power Version 3.1” program for the comparison between the WEP-CARE and TAU groups at the 3MFU assessment with regard to the primary outcome variable. The expected pre-post effect size in the WEP-CARE group is based on previously reported results of our pilot study (anxiety reduction *d* = 2.06) [21] and a meta-analysis on the effectiveness of iCBT to reduce anxiety (*d* = 0.77–1.11) [23]. Due to the possibility of spontaneous remission or improvement in anxiety symptoms through TAU, we conservatively assumed an effect size of *d* = 0.80 in the TAU condition, similar to the RCT conducted by Goldbeck et al. [34]. Assuming those effect sizes, utilizing the power of a statistical test of 80.0% and α < 0.05 (2-tailed), 52 participants (26 per group) are required for the analysis using a t-test. For an intention to treat analysis (ITT), the number of cases for dropouts does not have to be adjusted, but any violations of the normal distribution, effects due to imputation methods in the case of missing values, and possible residual effects must be considered in the planning. Therefore, the required number of cases is increased to *n* = 35 per group (total *N* = 70).

### Statistical methods

ITT analyses were performed to test our hypotheses. Since Little’s missing completely at random (MCAR) test indicated that our data were missing completely at random across all dimensional outcome measures and assessments (baseline, 3MFU and 6MFU, except for depression at 6MFU), the expectation-maximization (EM) algorithm [35] was used to account for missing data separately for each instrument (GAD-7, FoP-Q-SF, PHQ-9, ULQIE and CHIP-D) and condition (WEP-CARE and TAU). The group differences, with measurement time point (baseline, 3MFU) as a repeated-measures independent variable, condition (WEP-CARE, TAU) as a between-group independent variable, and anxiety, fear of disease progression, depression, QoL and coping as the dependent variables, were tested through repeated-measures analyses of variances (ANOVAs). Anxiety at 3MFU was a priori determined the primary outcome variable. Other dependent variables were analysed in an exploratory manner. The interaction term of time and group was used as an indicator of superiority of WEP-CARE over the TAU condition. The WEP-CARE group was additionally followed up three months after the treatment. Bonferroni correction was used for multiple comparisons. Cohen’s effect size d [36] was calculated for within-group pre-3MFU and pre-6MFU comparisons and to estimate the between-group effect size at 3MFU, adjusted for baseline values [37]. Furthermore, in the case of superiority of the WEP-CARE condition, a significant difference between baseline and 6MFU scores (if suggesting significantly improved mental health at 6MFU) was used as an indicator of the sustainability of the WEP-CARE effects, whereas a significant difference between 3MFU and 6MFU was an indicator of further improvement or decline of mental health. Clinically elevated anxiety, fear of disease progression and depression in parents were reported based on proposed cut-offs for GAD-7, FoP-Q-SF and PHQ-9. Absolute and relative frequencies for user satisfaction were calculated. The significance level for all statistical tests was set at *p* < .05 (2-tailed). Statistical analyses were performed using IBM SPSS Statistics 28.

## Results

Out of 74 study participants, 48 were assessed at 3MFU and 15 at 6MFU. Only data from the WEP-CARE participants were analysed at 6MFU since TAU participants received WEP-CARE after 3MFU (see Fig. 1). Four participants in each group reported receiving psychotherapy/counselling during study participation. Three participants in the TAU and five in the WEP-CARE condition reported utilizing other psychosocial interventions. There was no statistically significant difference between the groups regarding an additional psychotherapy/counselling (*p* = 1.000) or psychosocial interventions (*p* = .436), reported at 3MFU. However, five participants in the TAU and no one in the WEP-CARE condition reported taking medication at 3MFU (*p* = .053).

### Primary outcome

The average parental anxiety symptom level, assessed with the GAD-7, was moderate (≥ 10) at the time of study admission for the whole study sample and both conditions (see Table 1). Participants randomly assigned to the WEP-CARE condition reported on average mild anxiety after completing the intervention and three months later. In contrast, participants in the TAU condition still presented moderate anxiety after the waiting period. Furthermore, large effect sizes were found at posttreatment and three months later for the WEP-CARE group, whereas a small effect size was found after completing TAU. Repeated-measures ANOVA indicated both a significant time effect and a significant time-group interaction, suggesting a significant reduction in parental anxiety symptoms in the whole study sample and superiority of the WEP-CARE intervention. Furthermore, a significant difference between baseline and 6MFU indicated sustainability of the WEP-CARE effects three months posttreatment (see Table 2).

**Table 2:** Primary and secondary outcomes based on ITT analyses with EM: means, standard deviations, effect sizes by group (WEP-CARE, TAU) and assessment time point (baseline, 3MFU, 6MFU) and results of repeated measures ANOVAs within two assessment time points (baseline and 3MFU) and the group variable (WEP-CARE, TAU)

Outcome	WEP-CARE					TAU		ANOVA		Controlled effect size d
	3MFU: M (SD)	Effect size d between baseline and 3MFU	6MFU: M (SD)	Difference between baseline and 6MFU	Effect size d between baseline and 6MFU	3MFU: M (SD)	Effect size d between baseline and 3MFU	Time (baseline,3MFU)	Interaction (time X group)	
GAD-7	7.81 (4.51)	0.83	6.47 (4.42)	3.90; *p* < .001	1.13	10.67 (4.39)	0.33	*F*1,206 = 35.68; *p* < .001	*F*1,35 = 6.13; *p* = .016	0.49
FoP-Q-SF	33.90 (9.65)	0.58	30.07 (6.47)	8.24; *p* < .001	1.22	41.30 (7.97)	− 0.16	*F*1,119 = 6.54; *p* = .013	*F*1,331 = 18.23; *p* < .001	0.71
PHQ-9	8.27 (4.76)	0.51	6.07 (4.04)	*	1.00	11.19 (4.77)	− 0.08	*F*1,41 = 5.97; *p* = .017	*F*1,74 = 10.79; *p* = .002	0.60
ULQIE	55.39 (4.85)	0.65	54.41 (3.23)	**	0.57	53.44 (4.94)	0.66	*F*1,1608 = 13.11; *p* = .001	*F*1,2 = 0.016; *p* = .899	0.29
CHIP-D	27.58 (7.43)	0.45	30.29 (6.27)	− 6,78; *p* = .001	0.79	23.21 (11.47)	− 0.08	*F*1,90 = 2.70; *p* = .105	*F*1,233 = 7.02; *p* = .010	0.47

*Note*: ITT = intention to treat analysis; EM = the expectation-maximization algorithm; 3MFU = 3-month follow-up; 6MFU = 6-month follow-up

WEP-CARE = a web-based psychological support program for caregivers of children with chronic rare diseases; TAU = Treatment-As-Usual; GAD-7 = Generalized Anxiety Disorder; FoP-Q-SF = the Short Form of the Fear of Progression Questionnaire; PHQ-9 = the Patient Health Questionnaire; *ULQIE = the Ulm Quality of Life Inventory for Parents, raw scores were transformed linearly into a scale ranging from 0 to 100; CHIP-D = the Coping Health Inventory for Parents

* Since the data at 6MFU were not missing completely at random (MCAR), the difference was not tested

** Sustainability was not tested if time X group interaction was not significant

### Secondary outcomes

The average parental fear of disease progression, assessed with FoP-Q-SF, was above the clinical cut-off (≥ 34) at baseline for the whole study sample and both conditions (see Table 1). At 3MFU, only the TAU participants reported clinically relevant fear of disease progression, but not the WEP-CARE participants. A medium effect size was found at posttreatment and a large effect size three months later for the WEP-CARE group, whereas a small negative effect size was found after completing TAU. Repeated-measures ANOVA indicated both a significant time effect and significant time-group interaction, suggesting a significant reduction in parental fear of disease progression in the whole study sample and superiority of the WEP-CARE intervention. Furthermore, a significant difference between baseline and 6MFU indicated sustainability of the WEP-CARE effects three months posttreatment (see Table 2). The difference regarding the fear of disease progression level between 3MFU and 6MFU was also significant (3.45; *p* < .001), suggesting additional symptom decline during the posttreatment observational period.

An average parental PHQ-9 symptom score at baseline suggested moderate symptoms of depression (≥ 10) at the time of study inclusion for the whole study sample and both conditions (see Table 1). The participants reported on average mild symptoms of depression after completing the WEP-CARE intervention and at three months posttreatment. In contrast, the participants in the TAU condition still presented moderate symptoms of depression after the waiting period. A medium effect size was found at posttreatment and a large effect size three months later for the WEP-CARE group, whereas a small negative effect size was found after completing TAU. Repeated-measures ANOVA indicated both a significant time effect and a significant time-group interaction, suggesting a significant symptom reduction for the whole study sample and superiority of the WEP-CARE intervention (see Table 2). Since the PHQ-9 data at 6MFU were not MCAR, the EM algorithm could not be used to account for missing data; hence, the stability of the treatment effects or any changes at 6MFU could not be tested.

The average parental QoL, assessed with ULQIE, was good at all measurement time points for the whole study sample and both conditions compared with a normative sample of parents caring for chronically ill children (see Tables 1 and 2, [7]). A medium effect size was found between baseline and 3MFU, in both conditions, and at 6MFU. Repeated-measures ANOVA indicated a significant time effect but not a significant time-group interaction, suggesting a significant improvement in parental QoL in the whole study sample, without superiority of any condition (see Table 2).

A small to medium effect size was found for parental coping, assessed with the CHIP-D scale Maintaining Social Support, Self-Esteem, and Psychological Stability, at posttreatment. A medium to large effect size was found three months later for the WEP-CARE group, whereas a small negative effect size was found after completing TAU. Repeated-measures ANOVA indicated a significant time-group interaction but not a significant time effect, suggesting superiority of the WEP-CARE intervention. Furthermore, a significant difference between baseline and 6MFU indicated sustainability of the WEP-CARE effects three months posttreatment (see Table 2). A difference between 3MFU and 6MFU was also significant (− 2.71; *p* = .021), suggesting additional improvement in parental coping during the posttreatment observational period.

*N* = 20 (95.2%) out of 21 participants in the WEP-CARE condition reported feeling well informed about the process/content of the intervention. *N* = 19 (90.5%) rated the WEP-CARE intervention as helpful. All WEP-CARE participants (100.0%) agreed that internet-based writing therapy for parents with chronically ill children should be integrated into regular clinical care. If they had to choose, out of 20, two WEP-CARE participants (10.0%) would prefer psychological support online, five (25.0%) in a face-to-face setting and 13 (65.0%) found both options equally preferable. *N* = 17 (81.0%) WEP-CARE and 23 (85.2%) out of 27 TAU participants reported being able to easily get along with the menu navigation/operation on the UOC platform. All participants in the WEP-CARE (100.0%) and 25 (92.6%) in the TAU condition reported having confidence in personal data security on the platform.

The therapist satisfaction questionnaire was completed following each delivered WEP-CARE intervention. Data from the WEP-CARE condition and the pilot run were analysed together, since there were no changes in the intervention and the platform after the pilot run. The majority of the therapists evaluated different aspects of the WEP-CARE intervention positively (setting – internet-based writing therapy, WEP-CARE manual, supervision´s concept, platform design and usability, potential for implementation in daily routine), signaling willingness to continue to deliver internet-based writing therapy in the future (see Table 3). At the end, study therapists evaluated the intervention as follows: very good *n* = 3 (9.4%), good *n* = 19 (59.4%), satisfactory *n* = 9 (28.1%) and sufficient *n* = 1 (3.1%).

**Table 3:** Therapists’ satisfaction with the WEP-CARE intervention

Item	Response options			
	Strongly agree (%)	Agree (%)	Disagree (%)	Strongly disagree (%)
Internet-based writing therapy can be implemented in everyday life.	75.0	15.6	9.4	0.0
The WEP-CARE manual is very helpful.	50.0	46.9	0.0	3.1
The design of the WEP-CARE homepage is well-structured.	3.1	56.3	21.9	18.8
It was easy to get along with the menu navigation/operation on the UOC platform.	6.3	62.5	12.5	18.8
I really liked the design of the platform (colour, font, images, etc.).	25.0	31.3	25.0	18.8
I found the therapeutic work via the internet rather than in direct contact to be pleasant.	53.1	37.5	9.4	0.0
I found the contact with my patient, exclusively via text messages to be pleasant.	56.3	31.3	12.5	0.0
The amount of supervision received was appropriate.	90.6	9.4	0.0	0.0
I found the supervision in the written form after each writing task to be pleasant.	71.9	18.8	9.4	0.0
WEP-CARE has met my expectations.	25.0	62.5	9.4	3.1
I would continue to deliver internet-based writing therapy in the future.	84.4	15.6	0.0	0.0

*Note*: *N* = 32

WEP-CARE = a web-based psychological support program for caregivers of children with chronic rare diseases; UOC = Ulm Online Clinic

## Discussion

The purpose of this study was to further evaluate WEP-CARE within a sample of parents caring for children suffering from a wide range of different chronic RDs. Furthermore, since the efficacy of WEP-CARE has been investigated thus far only in the context of a pilot study, we aimed to compare WEP-CARE with TAU within an RCT design. The TAU condition represents the typical situation of the underserved population of parents caring for children with RDs in the current German (mental) health care system [18, 20]. Our RCT builds on prior findings of the efficacy of iCBT for different psychological problems, particularly anxiety and depression [22, 23].

In line with the pilot study [21], our primary outcome, parental generalized anxiety symptoms, declined statistically and clinically significantly in the course of the WEP-CARE intervention, and this effect was sustained for three months posttreatment. Beyond that, our data suggested clear superiority of the WEP-CARE intervention compared with TAU at the end of the treatment/waiting period. A similar pattern of results was observed for parental fear of disease progression, including an additional symptom decline between posttreatment and the end of the follow-up observational period. Regarding parental depression symptoms, our results suggested statistically and clinically significant symptom reduction and the superiority of the WEP-CARE intervention. Similar to fear of disease progression, parental coping improved not only during the intervention, in comparison to TAU, but also after the end of the intervention. These findings indicate that participants, as expected, learned to cope with perceived fears and threats related to the RD of their child. Finally, regarding QoL, only significant improvement of the whole study sample was found, without any difference between the conditions. Hence, improvement of QoL was not greater in the WEP-CARE group, which may be due to ceiling effects, since the baseline level of QoL was on average good. Another possible explanation is the limited sensitivity of the QoL measure for changes.

Finally, the vast majority of participating parents and study therapists evaluated the WEP-CARE intervention and UOC positively. Hence, all of the parents agreed that interventions such as WEP-CARE should be integrated into regular clinical care. The majority of the therapists reported willingness to deliver internet-based writing therapy in the future. Our results are in line with recent meta-analyses on iCBT that found overall participants´ satisfaction with the treatment across studies [22, 38].

## Conclusions

Altogether, our results confirmed that WEP-CARE is feasible for parents caring for children with a wide range of different chronic RDs, efficacious in reducing distress, improving coping and superior to usual care available in German psychosocial and psychiatric landscapes. Furthermore, our results suggest either stability of the treatment gains or further improvements over the posttreatment observational period across different parental mental health outcomes. Hence, WEP-CARE has the potential to contribute to closing the supply gap in treating families with children suffering from RDs, as it provides a scalable intervention that can be delivered to caretakers of children with chronic RDs independent of their place of residence.

Beyond that, this RCT provided important new knowledge regarding the efficacy of iCBT for different mental health problems, particularly anxiety and depression.

## Limitations and future directions

In addition to the impossibility of blinding the participants and therapists for the treatment, the absence of an active control group did not allow us to compare WEP-CARE with a comparator comprising the same dosage of attention. The lack of a control group during the posttreatment observational period means that the possibility that the long-term efficacy may be partially attributable to spontaneous remission cannot be ruled out. Furthermore, due to the predominance of well-educated, internet-savvy mothers in our study sample, the findings should only be generalized with great caution. The requirement of internet access could be an additional obstacle for families with low income. Our study sample consists of selected parents participating in a clinical study, which may have led to an overrepresentation of help-seeking parents with their own clinically relevant mental health problems. Limitations of self-report measures of psychological symptoms need to be mentioned. Finally, although comparable to the drop-out rates of other web-based psychotherapeutic interventions, a high rate of parents who did not start or complete the intervention should be acknowledged [39–41].

Future studies with larger and more representative community samples should be conducted by independent research groups, including follow-ups with lags of one year or more and active comparators during this observational period. Additionally, future studies should further investigate the impact of WEP-CARE on (1) parental mental health (including diagnostic status assessed with clinical interviews); (2) quality of communication in the family (particularly with child with an RD); (3) the ill child’s mental health; (4) RD treatment adherence and response and (5) use of health services - reduction of health-related costs. Finally, since only “living together with a partner” was associated with treatment adherence, further factors contributing to nonadherence to iCBT, such as lack of personal contact with a therapist, lack of time due to consuming demands associated with the child’s illness, the high workload and daily treatment routine in general, text-content complexity, discrepancy between participants’ expectations of the treatment process and the actual experiences of it, and benefits of new technologies, should be addressed in future studies [21, 40, 42].

### Supplement 1: Ulm Online Clinic

Ulm Online Clinic (UOC) is an online platform developed at Ulm University, Department of Child and Adolescent Psychiatry/Psychotherapy, for caring out internet-based projects for support and coping with chronic physical illnesses and stressful life events [25]. UOC is liable to strict data protection conditions, including anonymous registration, password protected access and secure data storage on a server based in Germany [25]. The entire data collection within the context of the RCT, as well as WEP-CARE intervention, took place via the secure internet platform UOC, protected against unauthorized access [25]. Communication fora was developed for communication between 1) study participants and therapists and 2) study therapists and their supervisors. All communication within the frame of UOC is cryptographically encrypted [25]. Conformity to the data protection regulations was confirmed for this study via approval from the relevant data protection authority at Ulm University.

## Data Availability

The data that support the findings of this study are not openly available due to reasons of sensitivity and are available from the corresponding author upon reasonable request. Data are located in controlled access data storage at Ulm University.

## References

[1] Richter T, Nestler-Parr S, Babela R, Khan ZM, Tesoro T, Molsen E (2015). Rare disease terminology and definitions—a systematic global review: report of the ISPOR rare disease special interest group. Value Health.

[2] European Medicines Agency [EMA]. Medicines for rare diseases. 2021. https://www.ema.europa.eu/en/human-regulatory/overview/orphan-designation-overview. Accessed 08 September 2022.

[3] De Vrueh R, Baekelandt ERF, De Haan JMH (2013). Background paper 6.19 rare diseases.

[4] EURORDIS-Rare Diseases Europe. What is a rare disease? 2020. http://www.eurordis.org/about-rare-diseases. Accessed 08 September 2022.

[5] Eidt D, Frank M, Reimann A, Wagner TOF, Mittendorf T, Graf von der Schulenburg. J-M. Maßnahmen zur Verbesserung der gesundheitlichen Situation von Menschen mit seltenen Erkrankungen in Deutschland. 2009. https://www.bundesgesundheitsministerium.de/fileadmin/Dateien/5_Publikationen/Praevention/Berichte/110516_Forschungsbericht_Seltene_Krankheiten.pdf. Accessed 08 September 2022.

[6] Witt S, Schuett K, Wiegand-Grefe S, Boettcher J, Quitmann J (2023). Living with a rare disease - experiences and needs in pediatric patients and their parents. Orphanet J Rare Dis.

[7] Goldbeck L, Storck M (2002). Das Ulmer Lebensqualitäts-Inventar für Eltern Chronisch Kranker Kinder (ULQIE). Z für Klinische Psychologie Und Psychother.

[8] Kenny T, Bogart K, Freedman A, Garthwaite C, Henley SMD, Bolz-Johnson M (2022). The importance of psychological support for parents and caregivers of children with a rare disease at diagnosis. Rare Disease and Orphan Drugs Journal.

[9] Tutus D, Niemitz M, Fegert JM, Wiegand-Grefe S, Fegert JM (2023). Chronische Somatische Erkrankungen mit psychischer Beteiligung in Kindheit Und Jugendzeit. Psychiatrie und Psychotherapie Des Kindes- Und Jugendalters.

[10] Tutus D, Niemitz M, Fegert JM, Rassenhofer M (2021). E-mental-health-angebote für Eltern eines Kindes Mit Einer Seltenen Chronischen Erkrankung. Monatsschrift Kinderheilkunde.

[11] Besier T, Born A, Henrich G, Hinz A, Quittner AL, Goldbeck L (2011). Anxiety, depression, and life satisfaction in parents caring for children with cystic fibrosis. Pediatr Pulmonol.

[12] Smith J, Cheater F, Bekker H (2013). Parents’ experiences of living with a child with a long-term condition: a rapid structured review of the literature. Health Expect.

[13] Quittner AL, Goldbeck L, Abbott J, Duff A, Lambrecht P, Solé A (2014). Prevalence of depression and anxiety in patients with cystic fibrosis and parent caregivers: results of the International Depression Epidemiological Study across nine countries. Thorax.

[14] van Oers HA, Haverman L, Limperg PF, van Dijk-Lokkart EM, Maurice-Stam H, Grootenhuis MA (2014). Anxiety and depression in mothers and fathers of a chronically ill child. Matern Child Health J.

[15] Fidika A, Herle M, Herschbach P, Goldbeck L (2015). Fear of disease progression questionnaire for parents: psychometric properties based on a sample of caregivers of children and adolescents with cystic fibrosis. J Psychosom Res.

[16] Boettcher J, Boettcher M, Wiegand-Grefe S, Zapf H (2021). Being the pillar for children with rare diseases—a systematic review on parental quality of life. Int J Environ Res Public Health.

[17] Niemitz M, Schrader M, Carlens J, Hengst M, Eismann C, Goldbeck L (2019). Patient education for children with interstitial lung diseases and their caregivers: a pilot study. Patient Educ Couns.

[18] Boettcher J, Filter B, Denecke J, Hot A, Daubmann A, Zapf A, et al. Evaluation of two family-based intervention programs for children affected by rare disease and their families– research network (CARE-FAM-NET): study protocol for a rater-blinded, randomized, controlled, multicenter trial in a 2x2 factorial design. BMC Fam Pract. 2020;21. 10.1186/s12875-020-01312-9. 10.1186/s12875-020-01312-9 PMC7678588 33218310

[19] Tutus D, Plener PL, Niemitz M (2020). Qualitätskriterien internetbasierter kognitiv-behavioraler Interventionen für Kinder Und Jugendliche sowie deren Eltern—Ein systematisches review. Zeitschrift für Kinder- und Jugendpsychiatrie Und Psychotherapie.

[20] Besier T, Goldbeck L (2011). Anxiety and depression in adolescents with CF and their caregivers. J Cyst Fibros.

[21] Fidika A, Herle M, Lehmann C, Weiss C, Knaevelsrud C, Goldbeck L (2015). A web-based psychological support program for caregivers of children with cystic fibrosis: a pilot study. Health Qual Life Outcomes.

[22] Etzelmueller A, Vis C, Karyotaki E, Baumeister H, Titov N, Berking M (2020). Effects of internet-based cognitive behavioral therapy in routine care for adults in treatment for depression and anxiety: systematic review and meta-analysis. J Med Internet Res.

[23] Hedman E, Ljótsson B, Lindefors N (2012). Cognitive behavior therapy via the internet: a systematic review of applications, clinical efficacy and cost–effectiveness. Expert Rev PharmacoEcon Outcomes Res.

[24] Mundlos C. Den Menschen Mit Seltenen Erkrankungen Eine Stimme geben: ACHSE e. V. Der Internist (Berl). 2018;59(12):1327–34; 10.1007/s00108-018-0517-z. PMID:30377713. 10.1007/s00108-018-0517-z 30377713

[25] Tutus D, Plener PL, Niemitz M (2018). Ulmer Onlineklinik– Eine Plattform für internetbasierte psychodiagnostik und psychologische online-interventionsprogramme. PiD - Psychotherapie Im Dialog.

[26] Löwe B, Decker O, Müller S, Brähler E, Schellberg D, Herzog W, et al. Validation and standardization of the generalized anxiety disorder screener (GAD-7) in the general population. Med Care. 2008;266–74. 10.1097/MLR.0b013e318160d093. 10.1097/MLR.0b013e318160d093 18388841

[27] Spitzer RL, Kroenke K, Williams JB, Löwe B (2006). A brief measure for assessing generalized anxiety disorder: the GAD-7. Arch Intern Med.

[28] Kroenke K, Spitzer RL, Williams JB (2001). The PHQ-9: validity of a brief depression severity measure. J Gen Intern Med.

[29] Gräfe K, Zipfel S, Herzog W, Löwe B (2004). Screening psychischer Störungen Mit dem Gesundheitsfragebogen für patienten (PHQ-D). Diagnostica.

[30] Lange A, Schrieken B, Blankers M, Van de Ven JP, Slot M (2000). Constructie en validatie Van de Gewaarwordingenlijst (GL): Een Hulpmiddel Bij het signaleren van een verhoogde kans op psychosen. Dth: Kwartaalschrift voor Directieve therapie en Hypnose.

[31] Mehnert A, Herschbach P, Berg P, Henrich G, Koch U (2006). Fear of progression in breast cancer patients–validation of the short form of the fear of Progression Questionnaire (FoP-Q-SF). Zeitschrift für Psychosomatische Medizin Und Psychotherapie.

[32] Herschbach P, Berg P, Waadt S, Duran G, Engst-Hastreiter U, Henrich G (2010). Group psychotherapy of dysfunctional fear of progression in patients with chronic arthritis or cancer. Psychother Psychosom.

[33] McCubbin HI, McCubbin MA, Cauble E, Goldbeck L (2001). Fragebogen Zur Elterlichen Krankheitsbewältigung: coping health inventory for parents (CHIP)-deutsche version. Kindh Entwickl.

[34] Goldbeck L, Muche R, Sachser C, Tutus D, Rosner R (2016). Effectiveness of trauma-focused cognitive behavioral therapy for children and adolescents: a randomized controlled trial in eight German mental health clinics. Psychother Psychosom.

[35] Dong Y, Peng C-YJ (2013). Principled missing data methods for researchers. Springerplus.

[36] Cohen J (1988). Statistical power analysis for the behavioral sciences.

[37] Klauer KJ (2001). Handbuch Kognitives Training.

[38] Terpstra JA, van der Vaart R, van Beugen S, van Eersel RA, Gkika I, Erdős D, et al. Guided internet-based cognitive-behavioral therapy for patients with chronic pain: a meta-analytic review. Internet Interventions. 2022;100587. 10.1016/j.invent.2022.100587. 10.1016/j.invent.2022.100587 PMC9672957 36406977

[39] Hadjistavropoulos HD, Mehta S, Wilhelms A, Keough MT, Sundström C (2020). A systematic review of internet-delivered cognitive behavior therapy for alcohol misuse: study characteristics, program content and outcomes. Cogn Behav Ther.

[40] Johansson O, Michel T, Andersson G, Paxling B (2015). Experiences of non-adherence to internet-delivered cognitive behavior therapy: a qualitative study. Internet Interventions.

[41] Meyerowitz-Katz G, Ravi S, Arnolda L, Feng X, Maberly G, Astell-Burt T (2020). Rates of attrition and dropout in app-based interventions for chronic disease: systematic review and meta-analysis. J Med Internet Res.

[42] Ross J, Stevenson F, Lau R, Murray E (2016). Factors that influence the implementation of e-health: a systematic review of systematic reviews (an update). Implement Sci.

